# Surface Functionalized Magnetic Nanoparticles as a Selective Sorbent for Affinity Fishing of PPAR-γ Ligands from *Choerospondias axillaris*

**DOI:** 10.3390/molecules27103127

**Published:** 2022-05-13

**Authors:** Miaomiao Chi, Kunming Qin, Lei Cao, Min Zhang, Yingying Su, Xun Gao

**Affiliations:** 1 Jiangsu Key Laboratory of Marine Pharmaceutical Compound Screening, Jiangsu Ocean University, Lianyungang 222005, China; mmchi0818@163.com (M.C.); qinkm123@126.com (K.Q.); caolei970105@163.com (L.C.); minzhang20211021@163.com (M.Z.); s745530107@163.com (Y.S.); 2 Jiangsu Institute of Marine Resources Development, Jiangsu Ocean University, Lianyungang 222001, China; 3 Co-Innovation Center of Jiangsu Marine Bio-Industry Technology, Jiangsu Ocean University, Lianyungang 222001, China

**Keywords:** PPAR-γ, magnetic ligand fishing, *Choerospondias axillaris*, UHPLC-Q-Exactive orbitrap-MS/MS

## Abstract

Coronary heart disease (CHD), which has developed into one of the major diseases, was reported to be treated by the target of peroxisome proliferators-activate receptor γ (PPAR-γ). As a natural medicine long used in the treatment of CHD, there are few studies on how to screen the target active compounds with high specific activity from *Choerospondias axillaris*. To advance the pace of research on target-specific active compounds in natural medicines, we have combined magnetic ligand fishing and functionalized nano-microspheres to investigate the active ingredients of PPAR-γ targets in *Choerospondias axillaris*. The PPAR-γ functionalized magnetic nano-microspheres have been successfully synthesized and characterized by vibrating sample magnetometer (VSM), scanning electron microscopy (SEM), and transmission electron microscopy (TEM). The specificity, reusability, and reproducibility of the nano-microspheres were investigated with the help of the specific binding of rosiglitazone to PPAR-γ. In addition, the incubation temperature and the pH of the buffer solution in the magnetic ligand fishing were optimized to improve the specific adsorption efficiency of the analytes. Finally, with the aid of ultraperformance liquid chromatography plus Q-Exactive Orbitrap tandem mass spectrometry (UHPLC-Q-Exactive Orbitrap-MS/MS), the 16 active ligands including 9 organic acids, 5 flavonoids, and 2 phenols were found in the ethanolic extracts of *Choerospondias axillaris*. Therefore, the study can provide a successful precedent for realizing the designated extraction and rapid isolation of target-specific active ingredient groups in the complex mixtures.

## 1. Introduction

Coronary heart disease (CHD) occurs commonly in the middle-aged and elderly and remains the leading cause of morbidity and mortality worldwide [1]. A growing body of studies that have investigated the association between CHD and the outcome of incident obesity, diabetes, and hypertension has become available in recent years [2]. As a first-line drug for the treatment of CHD, thiazolidinedione has the most prominent cardiovascular effect on anti-atherosclerosis by triggering the target of peroxisome proliferators-activate receptor γ(PPAR-γ). Data gathered over the past few decades has illustrated that PPAR-γ participates in regulating anabolism and exerts modulatory actions in vascular cells to improve myocardial function [3,4]. Although the safety of rosiglitazone and thiazolidinediones, which may increase the risk of acute myocardial infarction, has received widespread attention, pioglitazone has been shown to reduce congestive heart failure and combined coronary events [5]. Therefore, there is a keen interest in developing selective modulators and agonists of PPAR-γ targets that could be useful for the treatment of CHD by increasing efficacy and reducing side effects.

*Choerospondias axillaris* has a long history in the clinical treatment of cardiovascular diseases [6]. Modern studies have shown that it has an obvious protective effect on acute myocardial ischemia, which is related to regulating the balance of various enzyme activities in vivo, inhibiting the occurrence of cell apoptosis after myocardial ischemia and affecting the expression of apoptosis-related genes [7,8,9]. Among them, the total flavonoids in *Choerospondias axillaris* produce a protective effect on regulating the expression profile of related proteins in the ischemic myocardium and have an obvious antagonistic effect on ADP-induced platelet aggregation. The organic acids in *Choerospondias axillaris* such as citric acid, malic acid, and succinic acid have been proved to resist myocardial ischemia-reperfusion injury [10]. Thus, flavonoids and organic acids in *Choerospondias axillaris* are considered to be the main components producing therapeutic effects, but there is little research on which specific compounds are acting therapeutically [11,12].

Ligand fishing has been recognized as a separation technique with significant prospects, especially in the discovery of bioactive metabolites from natural products. Immobilization of therapeutic targets on solid supports such as superparamagnetic particles has become an advanced bioanalytical tool for reliable and convenient identification of bioactive compounds in complex mixtures. Compared with the traditional separation methods, such as centrifugation [13], ultrafiltration [14], preparative chromatography [15], micro dialysis [16], and equilibrium dialysis [17], the technology of magnetic ligand fishing has been proved to isolate and extract the binders from complex matrixes in less time, at a lower cost, and with higher specificity [18,19,20,21].

Given the continuing increase in the number of patients with cardiovascular diseases worldwide, it is increasingly important to investigate the bioactive components of complex natural mixtures that act on PPAR-γ targets. In this study, we established a screening and identification method for potential ligands acting on PPAR-γ targets in *Choerospondias axillaris* with the help of the technology of magnetic ligand fishing and the analysis system of ultraperformance liquid chromatography plus Q-Exactive Orbitrap tandem mass spectrometry (UHPLC-Q-Exactive Orbitrap-MS/MS). Target–ligand components in complex ingredients can be rapidly screened by the integration of a variety of new technical methods, which is necessary for the isolation and identification of potential drugs with pharmacological effects from natural products.

## 2. Results and Discussion

### 2.1. Characterizations of GO@Fe_3_O_4_@SiO_2_ and GO@Fe_3_O_4_@SiO_2_-PPAR-γ Nanoparticles

**Figure 1:**
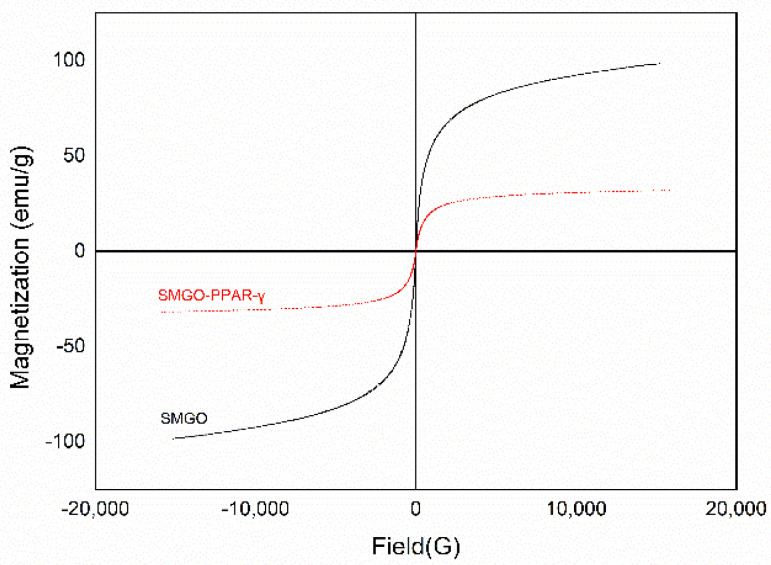
Magnetization curves of SMGO and SMGO−PPAR−γ at room temperature.

The magnetic properties of GO@Fe_3_O_4_@SiO_2_(SMGO) and GO@Fe_3_O_4_@SiO_2_-PPAR-γ (SMGO-PPAR-γ) were studied by using vibrating sample magnetometer (VSM) at room temperature, and the magnetization curves of the two nanoparticles were shown in Figure 1. It can be seen that the two nanoparticles showed superparamagnetic behavior with a saturation magnetization of 98.42 emu/g for SMGO and 31.75 emu/g for SMGO-PPAR-γ. Compared with SMGO, the saturation magnetization of SMGO-PPAR-γ decreased significantly by 66.67 emu/g, which attributed to the immobilization of the PPAR-γ enzyme on SMGO using the DNA double-strand. In spite of the decline of magnetic saturation, a good magnetization can still be maintained at room temperature, which provides conditions for effective magnetic separation.

**Figure 2:**
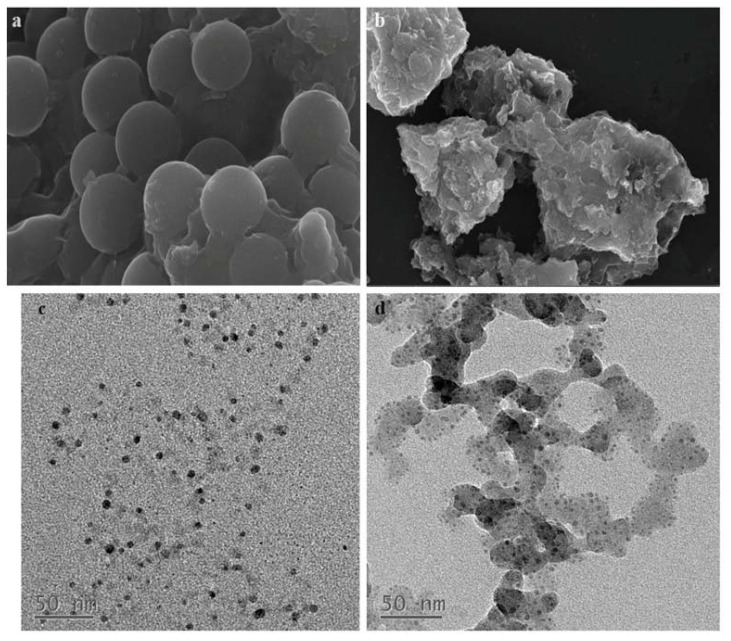
SEM images of SMGO (**a**), SMGO-PPAR-γ (**b**), TEM images of SMGO (**c**), and SMGO-PPAR-γ (**d**).

The morphology and structure of SMGO and SMGO-PPAR-γ were characterized by scanning electron microscopy (SEM) and transmission electron microscopy (TEM), and the results are shown in Figure 2. According to Figure 2a,b, we can see that the surface of SMGO shows a distinct spherical shape, while it is difficult to observe a smooth round spherical surface on the surface of SMGO-PPAR-γ. This is due to the filling of the SiO_2_ layer that originally created a gap to prevent agglomeration, causing the surface of SMGO-PPAR-γ to become rougher and fuller. The TEM images of SMGO and SMGO-PPAR-γ were shown in Figure 2c,d. It can be seen that the black spots previously uniformly anchored to the surface of the nanomaterial were replaced by distinct shell-like layers, which indicated some changes had been made on the surface of the SMGO. Thus, it can be concluded that PPAR-γ was successfully immobilized on the magnetic nanoparticles.

### 2.2. Assay Verification

Rosiglitazone is a highly selective PPAR-γ agonist, while fenofibrate acts as a PPAR-α agonist. The binding affinity of fenofibrate to PPAR-γ is at least 10-fold lower than that of PPAR-α. Therefore, rosiglitazone was chosen to investigate the properties of the nanoparticles by the binding differences of the different nanoparticles in this study.

Working pH and temperature directly affected the activity of PPAR-γ, while the washing step and dissociation solvent were indispensable for the dissociation of the specific bound component. Therefore, some essential experimental parameters including the pH of the buffer solution (from 5 to 9) and the incubation temperature (from 20 °C to 60 °C) were optimized during the process of magnetic ligand fishing, while washing times and dissociation solvent (methanol/PBS buffer solution) were investigated. The results in Table 1 reveal that the highest activity of immobilized PPAR-γ and the binding efficiency of rosiglitazone could be achieved in the following conditions, working the pH of the buffer solution at 6.0 and incubation temperature at 40 °C. Working buffer was used as washing solution three times and methanol was selected to dissociate the specific bound components.

**Figure 3:**
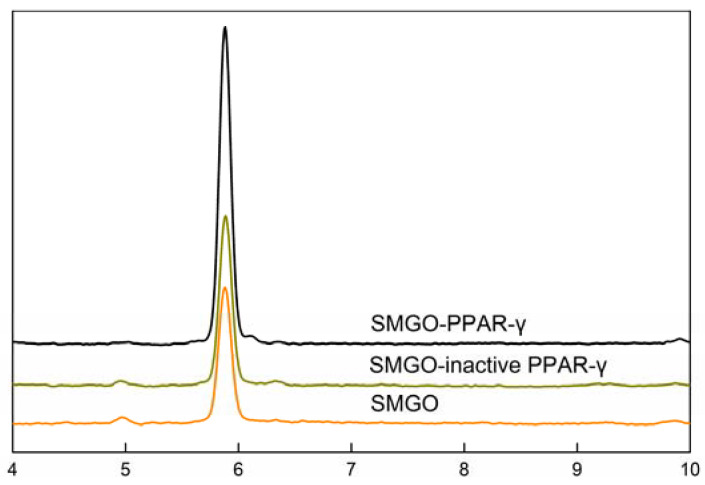
The HPLC chromatograms of rosiglitazone of ligand fishing assay eluent by SMGO-PPAR-γ SMGO-inactive PPAR-γ, and SMGO. Conditions: column: reversed phase symmetry C_18_ (250 mm × 4.6 mm i.d., 5 μm); mobile phase: NH_4_Ac in water (0.01 mol/L, pH = 6)-acetonitrile = 1:1; flow rate: 0.8 mL/min; column temperature: 35 °C; detector wavelength: 247 nm.

To explore the specificity of the nanoparticles, SMGO and SMGO-PPAR-γ were incubated with rosiglitazone solution, respectively. After dissociation with methanol, the supernatant was introduced into HPLC for analysis and the results are shown in Figure 3. The ability of binding rosiglitazone of SMGO-PPAR-γ nanoparticles was significantly higher than that of SMGO, while the ability decreased significantly after boiling at high temperature for 10 min to deactivate. Therefore, the results indicated that only SMGO-PPAR-γ can bind efficiently to rosiglitazone. Meanwhile, SMGO-PPAR-γ and SMGO-inactive PPAR-γ were incubated with a mixture of rosiglitazone and fenofibrate and the results showed that the ability of binding fenofibrate of SMGO-PPAR-γ nanoparticles was the same as SMGO-inactive PPAR-γ. 

### 2.3. Reusability and Reproducibility of Immobilized PPAR-γ

The stable activity of immobilized PPAR-γ is a key prerequisite for performing a series of binding–dissociation cycles during which the same amount of ligand is captured to obtain highly reproducible and accurate data. The stability of SMGO-PPAR-γ was examined by five consecutive binding–dissociation cycles, and the reproducibility of the material was examined by preparing three batches of SMGO-PPAR-γ simultaneously.

**Figure 4:**
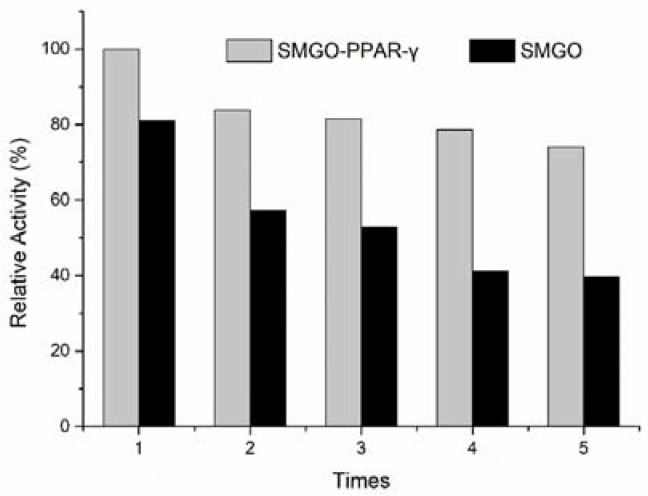
The relative activities of binding rosiglitazone in five consecutive association–dissociation cycles of ligand fishing assay eluent by SMGO-PPAR-γ and SMGO. Conditions: column: reversed phase symmetry C_18_ (250 mm × 4.6 mm i.d., 5 μm); mobile phase: NH_4_Ac in water (0.01 mol/L, pH = 6)-acetonitrile = 1:1; flow rate: 0.8 mL/min; column temperature: 35 °C; detector wavelength: 247 nm.

As shown in Figure 4, after five consecutive binding–dissociation cycles, the ability of binding rosiglitazone of SMGO-PPAR-γ nanoparticles was nearly 80% of the first cycle of binding–dissociation, while the ability of binding rosiglitazone of SMGO nanoparticles showed an obvious decreasing trend. Meanwhile, the three batches of SMGO-PPAR-γ nanoparticles showed similar ability of binding rosiglitazone (RSD < 3%). Therefore, the results showed that PPAR-γ grafted on the surface of SMGO can effectively improve the ability of binding rosiglitazone of the nanomaterials, slow down the declining trend after five cycles of binding–dissociation, and that SMGO-PPAR-γ nanoparticles have good stability and excellent reproducibility.

### 2.4. Identification of PPAR-γ Ligands from Choerospondias Axillaris by UHPLC-Q-Exactive Orbitrap-MS/MS

**Figure 5:**
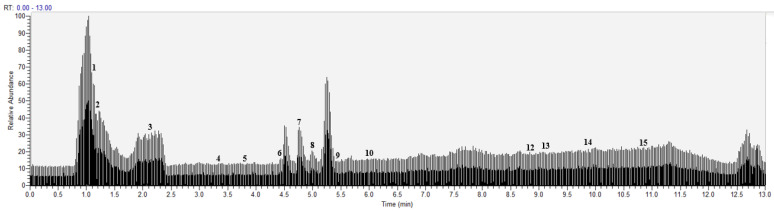
Base peak chromatogram of the elution solution of *Choerospondias axillaris* by UHPLC-Q-Exactive orbitrap-MS/MS in the negative mode.

**Figure 6:**
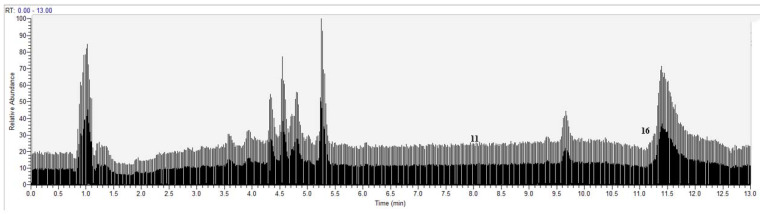
Base peak chromatogram of the elution solution of *Choerospondias axillaris* by UHPLC-Q-Exactive orbitrap-MS/MS in the positive mode.

Under the above chromatographic and mass spectrometric conditions, the elution of the target pendant extracted from *Choerospondias axillaris* was analyzed in the negative and positive mode. The results in the UHPLC-Q-Exactive Orbitrap-MS/MS system were shown in Figure 5 and Figure 6. According to the retention time of each component, mass spectrum information, and related references, 16 PPAR-γ ligands were preliminarily determined in the elution solution using the magnetic ligand fishing technique as listed in Table 2.

In the negative mode, all the compounds revealed deprotonated molecule [M−H]^−^ in the MS spectrum. Compounds **2**, **4**, **6**, **8**, **10**, and **12** were observed to have fragment ion peaks and similar peak intensities corresponding to the reference MS spectrum. The [M−162−H]^−^ ion in the MS spectra of compound **9** corresponded to the presence of hexose sugar and the [M−162−28−H]^−^ ion was observed in the fragments, which is attributed to the neutral loss of CO caused by the cleavage of carbonyl group. This compound was identified as hyperoside. The [M−18−H]^−^ ion in the MS spectrum of compounds **1** and **3** corresponded to the loss of dehydration, which is the typical fragment ion of phenolic acids. Therefore, compound **1** was characterized as quinine acid and compound **3** was identified as malic acid. The [M−44−H]^−^ ion observed in compounds **5**, **7**, and **15** corresponded to the neutral loss of CO_2_ caused by the cleavage of a carboxyl group from the [M−H]^−^. The [M−44−14−H]^−^ ion was observed in the fragments of compound **15**, which was attributed to the neutral loss of CH_2_ caused by the cleavage of a methylene group. Compounds **5**, **7**, and **15** were tentatively identified as protocatechuic acid (**5**), protocatechualdehyde (**7**), and pinocembrin (**15**) based on their fragmentation patterns. Compounds **13** and **14** exhibited [M−H]^−^ ion at *m*/*z* 255.23247 and *m*/*z* 283.26450, respectively, which were characterized as palmitic acid (**13**) and stearic acid (**14**) according to deprotonated molecular weights, retention time, and the reference [22]. In the positive mode, all the compounds revealed protonated molecule [M+H]^+^ in the MS spectrum. The [M−18+H]^+^ ion was observed in compounds **11** and **16**, which corresponded to the loss of H_2_O caused by the cleavage of dehydration from the [M+H]^+^. The fragment ion at *m*/*z* 137.05933 was observed in compound **11**, which is attributed to the characteristic and free fragments in balanophonin (**11**) [23]. According to the ion at *m*/*z* 281.24634 and the reference, compound **16** was characterized as linoleic acid [22].

### 2.5. Evaluate of PPAR-γ Ligands in Choerospondias Axillaris

With the help of magnetic ligand fishing and UHPLC-Q-Exactive Orbitrap-MS/MS, 16 ligands, including 9 organic acids, 5 flavonoids, and 2 phenols, were extracted and identified from *Choerospondias axillaris*. It has been reported that 12 of these ligands have varying degrees of activation or inhibition of PPAR-γ. According to the reports, compounds **1**, **6**, **9**, and **16** were able to increase the transcriptional activity of PPAR-γ in a dose-dependent manner using a reporter gene assay and a competitive ligand binding study [24,25]. Among them, it is particularly noteworthy that compound **16** increased gene expression of PPAR-γ target genes ap2, FATP, FAT, and adiponectin in white adipose tissue [26]. Meanwhile, compounds **5**, **10** and **12** were proven to stimulate the mRNA and protein expression of PPAR-γ in previous studies [27,28,29]. Among the organic acids, 10 μM of compounds **13** and **14** were reported to increase PPAR-γ response element activity 20% to 35% above basal levels, and the stimulation can affect insulin sensitivity and, hence, cardiovascular morbidity and mortality in humans [30]. In the gel densitometry scanning experiment of Prabhakar, compound **4** increased the expression of PPAR-γ 1.52-fold in 3T3-L1 adipocytes [31]. PPAR-γ is also a nuclear receptor linked to the anti-inflammatory response. Compound **8** can activate the transcription factor PPAR-γ of anti-inflammatory in mice, and compounds **3**, **11**, and **15** decreased the TNF-α level of inflammatory cytokine and thence inhibited platelet aggregation on myocardial ischemia/reperfusion injury [32,33,34,35]. Conversely, compound **7** suppressed expression of the adipogenesis-related proteins PPAR-γ in the Oil Red-O staining of Jung [36]. Compound **2** has been reported to possess a variety of pharmacological effects, including cardioprotective, antithrombotic, anti-inflammatory, and antibacterial, which make it primarily used as a co-administrator with other drugs [37]. According to the related reports, we can conclude that the compounds analyzed by UPLC-Q-Exactive Orbitrap MS/MS were consistent.

## 3. Materials and Methods

### 3.1. Materials and Reagents

Graphene oxide was purchased from the Nanjing Jicang Nano Technology Co., Ltd. (Nanjing, China). Human recombinant protein PPAR-γ was supplied by Cayman Chemical. The reagents of ethanol, acetonitrile, ammonium acetate, ethyl oxalyl monochloride, rosiglitazone, fenofibrate, 3-aminopropyltriethoxysilane (APTES), and 4-(4,6-dimethoxy-1,3,5-triazin-2-yl)-4-methylmorpholinium chloride (DMTMM) were purchased from Shanghai Aladdin Biochemical Technology Co., Ltd. The two complementary DNA single strands (A chain; TACTATCTATCATACTGCCCTTTCCCCC; B chain; ATGATAGATAGTATG) were purchased from ShengGong Bioengineering (Shanghai) Co., Ltd. Water was supplied by Wahaha (Hangzhou, China).

### 3.2. Instrumentation

The magnetite nanoparticles were characterized by transmission electron microscopy, scanning electron microscope, and vibration sample magnetometer. TEM micrographs were obtained by the JEOL JEM2100F system (Japan Electron Optics Laboratory, Tokyo, Japan). SEM microstructure of nanoparticles was recorded by the Hitachi S4800 system (Hitachi, Ltd., Tokyo, Japan). The magnetic properties of nanoparticles were investigated by a Lakeshore VSM7407 (Lake Shore Cryotronics, Inc., Westerville, OH, USA).

### 3.3. HPLC Analysis

The reversed-phase symmetry C_18_ (250 mm × 4.6 mm i.d., 5 μm, Waters, Milford, MA, USA) column was employed for HPLC analysis of rosiglitazone. The isocratic mobile phase consisted of solvent A (acetonitrile) and B (0.01 mol/L NH_4_Ac in water, pH = 6). The mobile phase ratio was 50:50 and the flow rate was 0.8 mL/min. The temperature was 35 °C and the chromatogram was acquired at 247 nm.

The reversed-phase symmetry C_18_ (250 mm × 4.6 mm i.d., 5 μm, Waters, Milford, MA, USA) column was employed for HPLC analysis of fenofibrate. The isocratic mobile phase consisted of solvent C (methanol) and D (water). The mobile phase ratio was 85:15 and the flow rate were 0.8 mL/min. The temperature was 30 °C and the chromatogram was acquired at 286 nm.

### 3.4. UHPLC-Q-Exactive Orbitrap-MS/MS Analysis

The chromatographic separation of the eluent was performed on an analytical Shim-pack XR-ODSII (75 mm × 3.0 mm i.d., 1.7 μm, Shimadzu Research Laboratory Co. Ltd, Kyoto, Japan). The UHPLC-Q-Exactive Orbitrap-MS/MS system mainly consisted of an Ultimate 3000 series ultrahigh performance liquid chromatography (Thermo Fisher Scientific, San Jose, CA, USA) and Q-Exactive Orbitrap tandem mass spectrometer (Thermo Fisher Scientific, San Jose, CA, USA) via an electrospray ionization interface (ESI). The mobile phase consisted of A (acetonitrile) and E (0.4% acetic acid in water) at 30 °C and the elution gradient is shown in Table 3. All the mobile phases were prepared daily.

Mass spectra were acquired in both positive and negative ion mode through full MS and higher energy collisional dissociation data-dependent MS/MS analysis. Full-scan high-resolution accurate mass data acquisition captures all sample data, enabling identification of untargeted compounds and retrospective data analysis without re-running the samples. 

Ion source parameters were shown as follows: spray voltage −3.0 kV (negative polarity) and 3.3 kV (positive polarity), sheath gas 35 arbitrary units, auxiliary gas 10 arbitrary units, capillary temperature 350 °C, S-lens RF level 55, auxiliary gas heater temperature 350 °C. Automatic gain control was set at 1e6, the maximum injection time at 50 ms, and the isolation window at 2.0 *m*/*z*. The collision energy was varied in the range of 20–40 eV and scan range *m*/*z* 60–900 to obtain representative product ion spectra. The mass tolerance window was set to 5 ppm for the two analysis modes (2002/657/EC). Data analysis and processing have been performed using the Xcalibur 4.1 software (ThermoFisher Scientific, Waltham, MA, USA).

### 3.5. Synthesis of PPAR -γ Magnetic Nano-Microspheres

The GO@Fe_3_O_4_@SiO_2_-NH_2_(SMGO-NH_2_) nanoparticles were synthesized according to our previous work [38]. Based on the previous report, some modifications were made to coat PPAR-γ on the GO layer [39].

Pre-cool the ethyl oxalyl monochloride for 20 min in 25 mL of anhydrous acetonitrile under an ice bath. A total of 0.10 g SMGO-NH_2_ was added, accompanied by stirring mechanically for 1 h under an ice bath and washed with ultrapure water after the reaction. Next, 30 mL of anhydrous ethanol and 10 mL of NaOH (0.25 mol/L) were mixed and the above nanoparticles were added, stirred mechanically at 60 °C for 1 h, and washed with ultrapure water three times. This was followed by 50 mL of 120 μM of condensation agent DMTMM and 20 μL of 100 μM B-chain DNA sequentially, stirred for 1 h at room temperature, and washed with ultrapure water. Then, 20 μL of 100 μM A chain DNA was added and mechanically stirred at 100 °C for 5 min. After the reaction was completed, the solution was cooled to room temperature and washed with ultrapure water and 10 mM PBS solution. Finally, dissolved in PBS buffer, PPAR-γ was bonded to B chain DNA under an ice bath for 3 h. After finishing the reaction, the SMGO-PPAR-γ was obtained after washing with ultrapure water and PBS buffer solution.

### 3.6. Magnetic Ligand Fishing

The powder is obtained by grinding the fruit of *Choerospondias axillaris*. A total of 5.0 g of powder were extracted with 500 mL of 75% (*v*/*v*) ethanol three times in parallel at 85 °C for 1.5 h. After filtering on three layers of gauze of the diameter of 4.5 mm, the extraction solutions were combined, freeze-dried, and dissolved to 2 mg/mL. The mixture of 20 mg of SMGO-PPAR-γ nano-microspheres and 2 mL of extraction solution was incubated at 40 °C for 30 min. The non-specific absorbents were removed by 10 mM PBS buffer solution (pH = 6.0) with the help of a magnet. Subsequently, the supernatant was obtained by incubating with 2 mL of methanol for 1 h to dissociate the extracted ligand. After filtering with 0.22 μm membrane, the supernatant was injected into UHPLC-Q-Exactive Orbitrap-MS/MS for analysis. 

## 4. Conclusions

In this study, we have proposed a new method based on SMGO-PPAR-γ ligand fishing for facile and rapid screening PPAR-γ ligands from complex natural products. UHPLC-Q-Exactive Orbitrap-MS/MS analysis was employed for structural elucidation. The proposed method was verified by selective PPAR-γ agonist, rosiglitazone, which indicated the method could successfully isolate PPAR-γ ligands and remove nonspecific binding. Another noteworthy advantage of the method was that immobilized PPAR-γ was stable, meaning it could perform multiple fishing, enhance sample throughput, and attain high reproducibility. Applied for the ethanolic extracts of *Choerospondias axillaris*, 16 ligands including 9 organic acids, 5 flavonoids, and 2 phenols were screened and identified. The results indicated that the developed method facilitates the discovery of biologically active compounds in natural products. 

## Figures and Tables

**Table 1:** Results of the experimental parameters during magnetic ligand fishing.

Category		Relative Activity (%)	Category		Relative Activity (%)	Category		Relative Activity (%)
Temp (degree)	25	40.1 ± 4.5	pH	5	13.6 ± 2.1	Dissociation solvent	Methanol	100 ± 0.2
	35	85.3 ± 3.8		6	100 ± 11.6		PBS	ND
	45	100 ± 0.2		7	61.1 ± 6.6	Washing times	2	199.2
	55	44.2 ± 3.8		8	54.7 ± 4.3		3	100.2
	60	27.8 ± 3.0		9	45.0 ± 5.0		4	99.5

**Table 2:** Identification of elution solution by UHPLC-Q-Exactive Orbitrap-MS/MS.

No.	Identification	Chemical Formula	t_R_ (min)	Ion Mode	Observe Mass (Da)	Error (ppm)	MS/MS	Confidential Levels
1	Quinine acid	C_7_H_12_O_6_	1.17	-	191.05528	−4.351	173.05528[M-H_2_O-H]^−^ 93.04486[M-2H_2_O-CO-2OH^−^-H]^−^	2
2	Succinic acid	C_4_H_6_O_4_	1.24	-	117.01888	−3.862	99.00845[M-H_2_O-H]^−^ 73.02924[M-CO_2_-H]^−^	1
3	Malic acid	C_4_H_6_O_5_	1.89	-	133.01387	−2.831	115.00314[M-H_2_O-H]^−^ 71.01357[M-H_2_O-CO_2_-H]^−^	2
4	Vanillic acid	C_8_H_8_O_4_	3.43	-	167.03423	−4.502	152.01108[M-CH_3_-H]^-^ 108.02119[M-CH_3_-CO_2_-H]^−^	1
5	Protocatechuic acid	C_7_H_6_O_4_	3.89	-	153.01872	−3.999	109.02916[M-CO_2_-H]^−^	2
6	Catechin	C_15_H_14_O_6_	4.44	-	289.07178	0.064	245.08138[M-CO_2_-H]^−^ 203.07051[M-C_4_H_6_O_2_-H]^−^	1
7	Protocatechualdehyde	C_7_H_6_O_3_	4.77	-	137.02383	−4.268	109.02925[M-CO-H]^−^ 93.03424[M-CO_2_-H]^−^	2
8	Caffeic acid	C_9_H_8_O_4_	5.00	-	179.03555	3.172	135.04459[M-CO_2_-H]^−^	1
9	Hyperoside	C_21_H_20_O_12_	5.42	-	463.08972	3.284	300.02646[M-C_6_H_11_O_5_-H]^−^ 271.02484[M-C_6_H_12_O_5_-CO-H]^−^	2
10	Taxifolin	C_15_H_12_O_7_	5.99	-	303.04892	−6.949	285.04056[M-H_2_O-H]^−^ 125.02381[M-C_9_H_6_O_4_-H]^−^	1
11	Balanophonin	C_20_H_20_O_6_	7.90	+	357.13586	7.267	339.12207[M-H_2_O+H]^+^ 320.11118[M-2H_2_O+H]^+^ 137.05933[C_8_H_8_O_2_+H]^+^	2
12	Naringenin	C_15_H_12_O_5_	8.96	-	271.06107	−0.468	151.00325[M-C_6_H_7_O-2CH_3_-H]^−^	1
13	Palmitic acid	C_16_H_32_O_2_	9.21	-	255.23247	−1.894	/	3
14	Stearic acid	C_18_H_36_O_2_	9.73	-	283.26450	0.411	/	3
15	Pinocembrin	C_15_H_12_O_4_	10.74	-	255.06564	−2.518	227.07065[M-CO_2_-H]^−^ 213.05502[M-CO_2_-CH_2_-H]^−^	2
16	Linoleic acid	C_18_H_32_O_2_	11.03	+	281.24634	−4.149	263.23615[M-H_2_O+H]^+^	2

Confidential level 1: Compounds that matched reference standard. Confidential level 2: Compounds that matched robust spectral or the literature. Confidential level 3: Compounds that speculated.

**Table 3:** The elution gradient of UHPLC-Q-Exactive Orbitrap-MS/MS.

**Time (min** **)**	0	2	3	7	9	10	10.01	12
**A (%)**	5	15	25	45	60	75	5	5
**C (%)**	95	85	75	55	40	25	95	95

## Data Availability

Not applicable.
